# Acknowledgement to Reviewers of *Dieseases* in 2014

**DOI:** 10.3390/diseases3010001

**Published:** 2015-01-09

**Authors:** Dieseases Editorial Office

**Affiliations:** MDPI AG, Klybeckstrasse 64, CH-4057 Basel, Switzerland

The editors of *Diseases* would like to express their sincere gratitude to the following reviewers for assessing manuscripts in 2014:

Alexandraki, Krystallenia I.
Applemelk, Ben
Barry, Meagan A
Bauer, Natalie
Blaner, William
Bodet, Charles
Burger, Charles D.
Carrel, Thierry
Chan, Anthony WH
Chan, Stephen L.
Chiu, Hsin-Hui
De Cooman, Lien
D'elios, Mario M.
Dellegrottaglie, Santo
den Hollander, Wouter J.
Ducharme, Anique
Eladari, Dominique
Emoto, Noriaki
Figura, Natale
Fraga, Garth
Gaetano, Carlo
Genta, R. M.
Groepenhoff, Herman
Guarner, Jeannette
Guignabert, Christophe
Han, Ying
Harada, Kenichi
Houghton, Michael
Johnson, Martin K.
Karanikolos, Marina
Kari, Fabian A.
Ko, Christine
Lange, Tobias J.
Li, Wei
Liou, Jyh-Ming
Liu, Chun-Jie
Lu, Da-Bing
Markovska, Rumyana
Moriyama, Mitsuhiko
Morris, Shaine
Naeije, R.
Newbigging, Susan
Paulin, R.
Paunescu, Teodor G.
Piersigill, Alessandra
Rautelin, Hilpi
Robinson, Karen
Samoutis, George
Santucci, Annalisa
Schneider, Barbara
Sharma, Amar Deep
Song, Howard K.
Song, Huan
Sugimoto, Mitsushige
Takeda, Norifumi
van der Veerdonk, Mariëlle C
Wells, J. Michael
Wort, John

